# Changing expression profiles of long non-coding RNAs, mRNAs and circular RNAs in ethylene glycol-induced kidney calculi rats

**DOI:** 10.1186/s12864-018-5052-8

**Published:** 2018-09-10

**Authors:** Yanan Cao, Xiaowei Gao, Yue Yang, Zhi Ye, E. Wang, Zhitao Dong

**Affiliations:** 1 Department of Anesthesiology, Xiangya Hospital, Central South University, Xiangya Road 87#, Changsha, Hunan 410008 People’s Republic of China; 2 Department of Urology, Second Xiangya Hospital, Central South University, Changsha, Hunan 410011 People’s Republic of China

**Keywords:** Kidney stone, Calcium oxalate, Long non-coding RNAs, mRNAs, circRNAs

## Abstract

**Background:**

To explore long non-coding RNA (lncRNA), mRNA and circular RNA (circRNA) expression profiles and their biological functions in the pathogenesis of kidney stones in ethylene glycol-induced urolithiasis rats.

**Results:**

The expression of 1440 lncRNAs, 2455 mRNAs and 145 circRNAs was altered in the kidneys of urolithiasis rats. GO and KEGG biological pathway analysis were performed to predict the functions of differentially expressed lncRNAs, circRNAs and co-expressed potential targeting genes. Co-expression networks of lncRNA-mRNA and circRNA-miRNA were constructed based on correlation analysis between differentially expressed RNAs. mRNAs coexpressed with lncRNAs were involved in many kidney diseases, e.g., Ephb6 was associated with the reabsorption ability of the kidney. Arl5b was associated with the dynamic changes in the podocyte foot process in podocyte injury. miRNAs co-expressed with circRNAs, such as rno-miR-138-5p and rno-miR-672-5p, have been proven to be functional in hypercalciuria urolithiasis.

**Conclusion:**

The expression profile provided a systematic perspective on the potential functions of lncRNAs and circRNAs in the pathogenesis of kidney stones. Differentially expressed lncRNAs and circRNAs might serve as treatment targets for kidney stones.

**Electronic supplementary material:**

The online version of this article (10.1186/s12864-018-5052-8) contains supplementary material, which is available to authorized users.

## Background

Kidney stones are a common chronic and systemic disease among Chinese adults, and currently, approximately one in 17 adults are affected [1, 2]. Kidney stone patients usually present with pain and urinary tract infection, which can lead to chronic renal disease and even kidney function loss [3, 4]. Approximately 80% of kidney stones are composed of calcium oxalate, calcium phosphate, or both [5]. Efforts have been made to study the molecular mechanisms of nephrolithiasis, but more exploration is needed based on advances in bioinformatics.

Long non-coding RNA (lncRNA) is a class of non-coding transcripts longer than 200 nucleotides. Studies have reported that lncRNAs are involved in many kidney diseases, including renal carcinoma [6], renal fibrosis [7], acute kidney injury [8], and calcium oxalate-induced kidney damage [9]. It has been found that LncRNA CHCHD4P4 inhibited cell proliferation and promoted the epithelial-mesenchymal transition in kidney damage and fibrosis caused by calcium oxalate crystallization and deposition [9].

Circular RNA (circRNA) is another class of non-coding RNA composed of a continuous, covalently closed loop [10]. Circular RNA s may function similarly to regulate the activity of other microRNAs. Circular RNAs may bind RNA-binding proteins or even base pair with RNAs, resulting in the formation of large RNA-protein complexes. For example, circHIAT1 suppressed androgen receptor-enhanced clear cell renal cell carcinoma cell migration and invasion [11]. Similar to lncRNAs, the expression profiles of circRNAs are specific among different pathological processes, indicating a possible regulatory function.

To date, little is known about the functions of lncRNAs and circRNAs in the pathological processes of kidney stones. In our study, we performed RNA sequencing on the expression profiles of lncRNAs, mRNAs, and circRNAs in rats with kidney stones. We also conducted GO and KEGG pathway analyses and constructed co-expression networks. Our findings might illuminate a novel mechanism of nephrolithiasis pathogenesis and provide new targets for the prevention and treatment of kidney stones.

## Methods

### Animal care

All animal experiments were performed with adult male Sprague-Dawley (SD) rats (250–300 g, obtained from the Laboratory animal center of Central South University Changsha, China) in accordance with guidelines according to the Central South University of Science and Technology Guide for the Care and Use of Laboratory Animals. All experimental animal procedures were approved by the Institutional Animal Care and Use Committee of Xiangya Hospital of Central South University. The rats were maintained and habituated in a standard 12-h light-dark cycle with ad libitum access to food and water in a temperature- and humidity-controlled room, maintaining 22 °C ± 0.5 °C and a relative humidity of 40–60%.

### Kidney stone modeling experiments

Rats were randomly divided into a control group (*n* = 4) and kidney stone group (n = 4). The control group only received normal rat chow and sterile water for 28 days. The kidney stone group received drinking water with 1% EG (Sigma-Aldrich, Buchs, Switzerland) for 28 days.

### Analysis of renal function and histology

Twenty-four-hour (24-h) urine of rats in each group was collected on the 27th and 28th days using metabolic cages. Food and water were available in the cages during experimentation. The data for total urine were collected. For analysis of urine, 1 mL of urine was collected in a centrifugal tube and centrifuged at 2500 rpm for 5 min to obtain urine. Urine was stored at − 20 °C. The specific gravity, pH, and concentrations of calcium, citrate, sodium, potassium, uric acid, creatinine and urea nitrogen of the urine were measured with an Auto Analyzer (Hitachi, 7170A, Japan).

The blood of rats in each group was collected on the 28th day. Rats were anesthetized via inhalation of sevoflurane. Blood was collected from the inferior vena cava in non-heparinized tubes and centrifuged at 3500 rpm for 15 min to obtain serum. Serum was stored at − 80 °C. The serum levels of potassium, sodium, chlorine, calcium, phosphorus, magnesium, uric acid, creatinine, urea nitrogen, and cystatin C were measured with an Auto Analyzer (Beckman Coulter, AU5821, CA, USA).

After the collection of blood, all rats were sacrificed via exsanguination, and bilateral kidneys were removed. One kidney per rat was fixed in 4% paraformaldehyde, dehydrated in ethanol solution, embedded into paraffin, sliced into 5-μm serial sections, stained with Hematoxylin and Eosin, and observed to detect CaOx crystals using a polarizing microscope. To evaluate the aggregation of CaOx deposits, 5 slides were selected randomly and observed in the microscopic field with a magnification of 10*40. The mean number of CaOx crystals counted and scored was calculated. The other kidney was frozen in liquid nitrogen and stored at − 80 °C for RNA sequencing analysis.

### RNA extraction

Total RNA was extracted from the kidney tissues of two groups using TRIzol reagent (Invitrogen, NY, USA) according to the manual instructions. Subsequently, total RNA was qualified and quantified using a NanoDrop spectrophotometer ND-1000 (Thermo Fisher Scientific, MA, USA).

### Construction of cDNA libraries and high-throughput sequencing

Total RNA was subjected to ribosomal RNA removal using a Ribo-Zero Magnetic kit (EpiCentre, WI, USA). RNAs were fragmented into short fragments using fragmentation buffer. The cDNA library was constructed using a TruSeq RNA sample Prep Kit (Illumina, CA, USA). Then, libraries were amplified through polymerase chain reaction on a cBot Cluster Generation System using the TruSeq PE Cluster Kit v3-cBot-HS (Illumina California, USA). Lastly, the libraries were sequenced using an Illumina HiSeq 2500 platform. Raw RNA-Seq data were flattened, and all subsequent analyses were performed using clean reads.

### Quantitative real-time PCR (qRT-PCR)

The results of high-throughput sequencing analysis were verified by qRT-PCR. The total RNA of the kidney stone group samples and control group samples was extracted using TRIZOL reagent (Invitrogen, NY, USA). A PrimeScript TM RT regent Kit (Takara Bio Inc., Otsu, Japan) was used to synthesize the cDNA. The qRT-PCR reactions were performed using All-in-One™ qPCR Mix (GeneCopoeia, Rockville, MD, USA) on the ABI Prism 7900 Sequence Detection System (Applied Biosystems, Foster City, USA) at 95 °C for 5 min, followed by 40 cycles of 95 °C for 10 s, 60 °C for 30 s, and 72 °C for 30 s. The primer sequences are listed in Table 1.

**Table 1:** qRT-PCR primer sequences

	Forward primer	Reverse primer
TCONS_00030209	AGTCAAATTCATGGGACTCG	GTGTGGTCAGGTCAATCTCTA
NONRATT009934.2	CAGATGACACCGTTAGGAT	TCAGGAAAGAAGGAAGCAACC
TCONS_00026280	CTGTGTGATTGAGGCAATCT	GTACTGGCTGGATTTCGTC
ENSRNOT00000000139	TTTGGTGTAGAGGATGACGAC	GGCTGCTGGAAGATGAAC
ENSRNOT00000006106	ACTGGACGTTCCATGAAAG	GCCAGCTCACAGGAAGTA
ENSRNOT00000003823	CTGTCCACAATGAGCTTCC	CAGCAGGGTGAATGACGA

The results were collected in three independent wells. Transcript levels of each lncRNA and mRNA were normalized by comparison with GAPDH using the 2^-∆∆CT^ method.

### Functional analysis

GO analysis and KEGG pathway analyses were conducted to predict the potential functions of DEmRNAs and DEcircRNAs. GO analysis was performed to annotate genes and gene products with terms from three aspects: biological process (BP), cellular component (CC), and molecular function (MF). KEGG pathway analysis was conducted to predict the molecular interactions and reaction networks associated with differentially expressed genes. Data were analyzed by two-sided Fisher’s exact test, and the FDR (false discovery rate) was calculated to correct the -log10 (*P* value). A cutoff of -log10 < 0.05 was set for statistical significance.

### Co-expression of lncRNAs/mRNAs and function prediction

The function of lncRNAs is forecasted according to annotations of the function of the coexpressed mRNAs. A DElncRNAs-DEmRNA co-expression network was constructed to explore the connection between lncRNAs and mRNAs in the pathogenesis of kidney stones. The Pearson correlation coefficient (PCC) was calculated between DElncRNAs and DEmRNAs. |PCC| no less than 0.8 and a *P* value no more than 0.05 were retained for further network construction.

Genomic localizations of the paired lncRNAs and mRNAs were identified for cis prediction. The co-expression nearby gene, which is less than 100 kb upstream or downstream from the lncRNA, can act as the potential target regulated by the lncRNA in a cis manner, while a trans-regulator is one that does not meet this criterion. The RIsearch-2.0 software was used to identify target genes in trans, with the parameter set as the base number of direct interactions between lncRNA and mRNA≥10 and free energy≤ − 50.

### Analysis of the circRNA-miRNA interaction network

The networks among circRNAs and miRNAs were predicted based on miRanda, with a maximum binding free energy of − 20. Cytoscape3.5.1 was used to display the circRNA-miRNA networks.

### Statistical analysis

qRT-PCR data were presented as the mean ± standard deviation (SD). Student’s t-test was used to determine the differences between groups, and *P* < 0.05 was regarded statistically significant. GraphPad Prism 6 Software (GraphPad, La Jolla, CA) was used for all statistical analyses.

## Results

### Urine parameters

As shown in Table 2, the specific gravity of the kidney stone group was significantly decreased compared to the normal group. This trend was also observed for pH. However, urinary output significantly increased in the kidney stone group. There was no difference in the concentration of urinary sodium, potassium, and phosphate. Urinary calcium, uric acid, urine creatinine and urea nitrogen were significantly increased in the kidney stone group.

**Table 2:** Effects of 1% ethylene glycol on urine, serum, and kidney parameters in normal rats and CaOx rats

Parameters	A	B	*P* value
Urine			
Specific Gravity	1.04 ± 0.01	1.02 ± 0.01	*
pH	7.48 ± 0.1	6.58 ± 0.17	*
Urinary output	41.75 ± 3.86	56.5 ± 3.42	**
Calcium (mmol/L)	1.33 ± 0.2	2.46 ± 0.29	**
Sodium (mmol/L)	82.33 ± 12.89	83.18 ± 7.69	ns
Potassium (mmol/L)	9.92 ± 1.01	9.76 ± 1.3	ns
Phosphate (mmol/L)	89.18 ± 9.94	79 ± 14.11	ns
Uric acid (mmol/L)	166.4 ± 9.69	781.55 ± 119.49	**
Creatinine (mmol/L)	560.4 ± 29.51	2608.55 ± 869.24	*
Urea Nitrogen (mmol/L)	99.69 ± 14.17	258.36 ± 33.57	**
Serum			
Potassium (mmol/L)	5.21 ± 0.83	5.02 ± 0.37	ns
Sodium (mmol/L)	144.55 ± 2.73	148.78 ± 0.94	*
Chlorine (mmol/L)	97.3 ± 1.1	96.93 ± 1.11	ns
Calcium (mmol/L)	2.93 ± 0.16	2.58 ± 0.1	**
Phosphorus (mmol/L)	2.83 ± 0.24	2.82 ± 0.11	ns
Magnesium (mmol/L)	0.97 ± 0.17	0.98 ± 0.16	ns
Uric acid (umol/L)	40.48 ± 12.28	168.03 ± 24.71	**
Creatinine (umol/L)	44.58 ± 2.94	57.25 ± 7.08	**
Urea Nitrogen (mmol/L)	4.92 ± 0.46	9.23 ± 0.59	**
Cystatin C (mg/L)	0.32 ± 0.02	0.37 ± 0.01	*

A and B groups represent normal and urolithiatic rats, respectively. The CaOx group received 1% EG in drinking water. Each column represents the mean ± SD for 4 rats

* represents *p* values < 0.05; ** represents *p* values < 0.01. ns represents no significance

### Serum parameters

As shown in Table 2, the serum potassium, chlorine, phosphorus, and magnesium were similar in the two groups. However, the concentrations of sodium and calcium were significantly increased in the kidney stone group. So were uric acid, urine creatinine, urea nitrogen and cystatin C.

### Histology results

**Figure 1:**
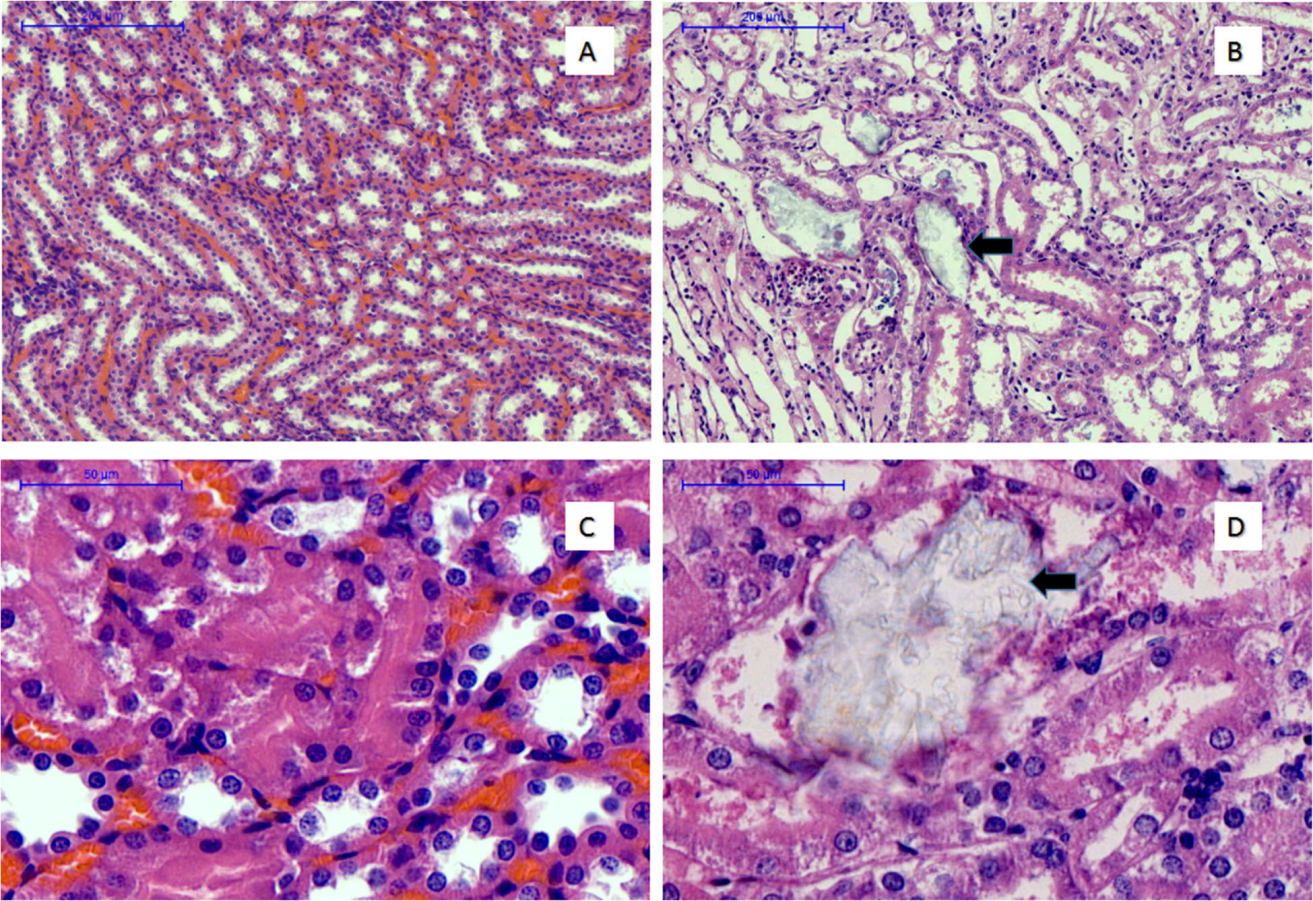
Representative microscopic images of kidney sections; Control Group showing the normal renal tubule (**a**, **c**). The urolithiasis group shows deposition of a large number of calcium oxalate crystals (black arrow, **b**, **d**)

Figure 1 suggests that in the 10× magnification and 40× magnification of the light microscope field, the renal parenchyma of normal rat had integrity, and there was no CaOx crystal formation. However, in the kidney stone group, the structure of the renal parenchyma was destroyed by abundant calcium oxalate crystals, which were colorless, with high refractivity (Arrow).

### Differentially expressed lncRNA, mRNA and circRNA profiles by RNA-sequencing

Kidney tissues obtained from the kidney stone group and control group were applied for RNA sequencing. After filtering the adaptor reads and low-quality tags, 9.0 × 10^7^, 8.8 × 10^7^, 8.9 × 10^7^ and 9.0 × 10^7^ clean reads were obtained from four samples from the kidney stone group, and 9.2 × 10^7^, 9.1 × 10^7^, 9.1 × 10^7^ and 9.1 × 10^7^ clean reads were generated from four corresponding control tissues (Additional file 1: Table S1). More than 88% of the raw reads per sample were clean reads. All clean reads were aligned with the human genome sequence GENCODE, Release 19. The mapped ratio in each sample was above 90% (Additional file 2: Table S2).

**Figure 2:**
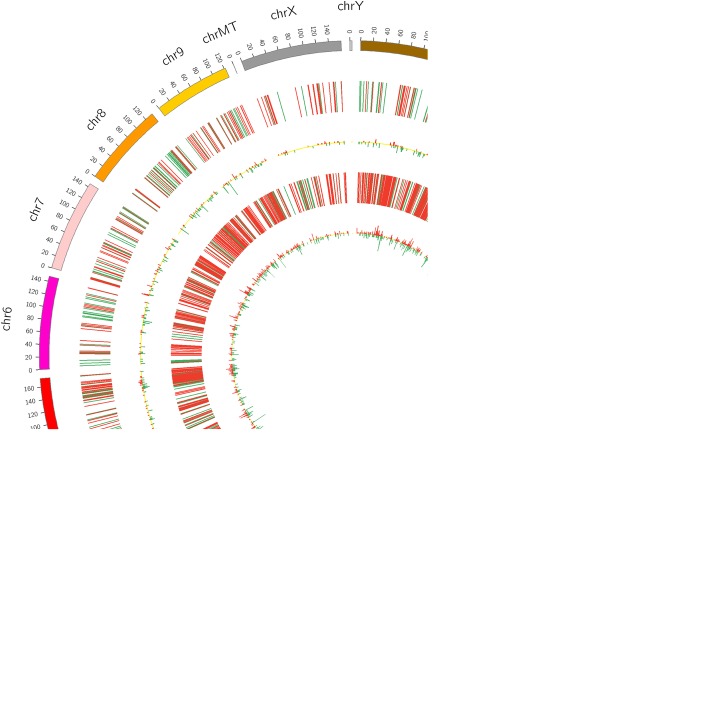
Circos plot showing lncRNAs and mRNAs on rat chromosomes. The outermost layer of the circos plot is a chromosome map of the rat genome. The largest and larger inner circles represents all differentially expressed lncRNAs detected by RNA-sequencing with fold change ≥2.0, *P* < 0.05 and FDR < 0.05. The increased or decreased lncRNAs have been marked with red or green bars, respectively, and bar heights in the larger inner circle represent numbers of differently expressed lncRNAs. The smaller and smallest inner circles represent all differentially expressed mRNAs detected by RNA-sequencing with fold change ≥2.0, P < 0.05 and FDR < 0.05. Increased or decreased lncRNAs are marked with red or green bars, respectively, and bar heights in the smallest inner circle represent numbers of differently expressed lncRNAs

**Figure 3:**
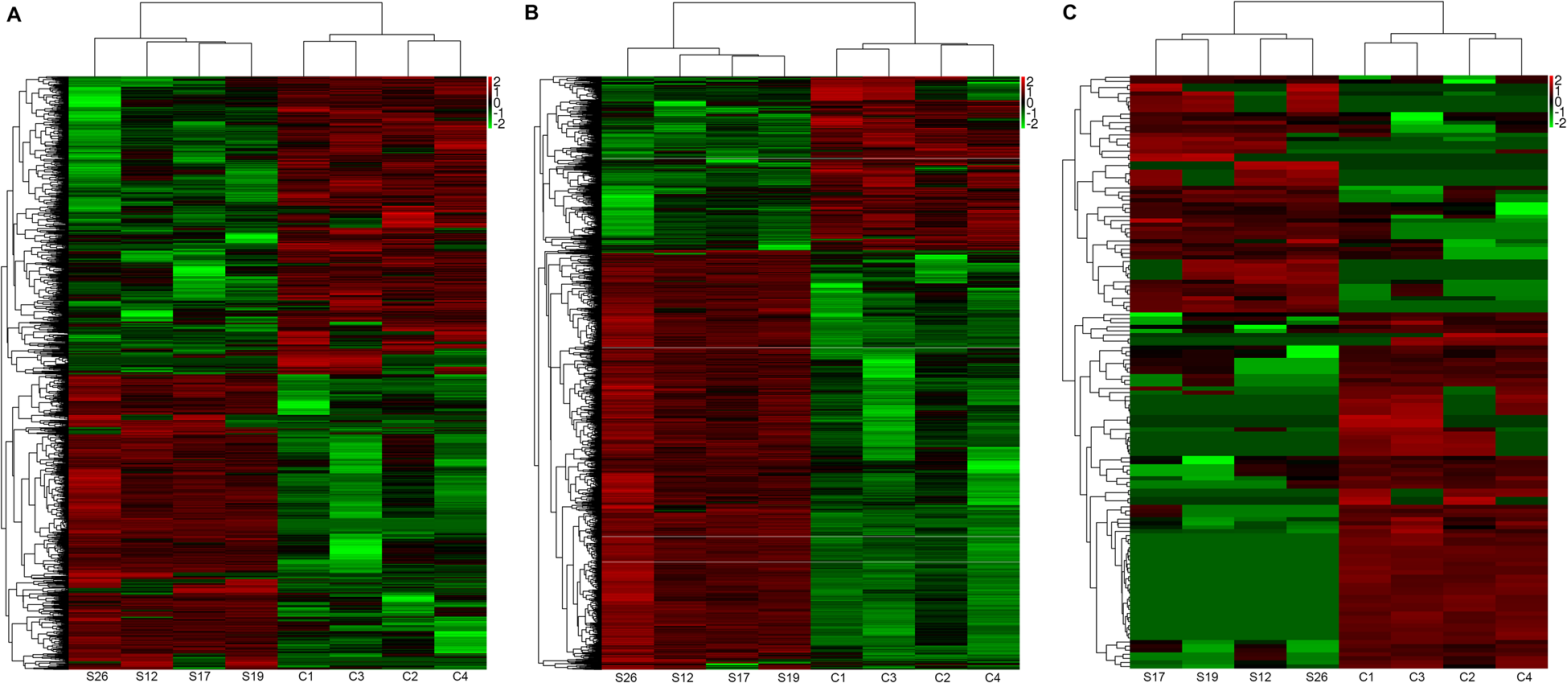
Heatmaps showing expression profiles of lncRNAs (**a**), mRNAs (**b**) and circRNAs (**c**). Heatmap showing DElncRNAs, mRNAs, and circRNAs from kidney tissues of urolithiasis rats compared to kidney tissues of control rats; row and column represent DElncRNA/DEmRNA/DEcircRNA transcripts and tissue samples. The color scale indicates log10 FPKM of expression levels of DElncRNAs/DEmRNAs/DEcircRNAs, and intensity increases from green to red, which indicate up- and down-regulation, respectivetly. S represents urolithiasis rats, and C represents control rats

In the present study, 1440 lncRNAs, 2455 mRNAs and 145 circRNAs were identified as remarkably differentially expressed, with |fold change| ≥2.0, *P* < 0.05 and FDR < 0.05. Both lncRNA and mRNA transcripts were found to be distributed on all chromosomes (Fig. 2). Among them, 711 lncRNAs were up-regulated, and 717 lncRNAs were down-regulated; meanwhile, 1732 and 723 mRNAs were up-regulated and down-regulated, respectively. There were 58 up-regulated circRNAs and 87 down-regulated circRNAs in four kidney stone tissues compared with the controls. Hierarchical clustering of the expression of the lncRNA, circRNA and mRNA showed obvious discrimination in kidney tissues between kidney stone rats and control rats (Fig. 3).

### Validation of deregulated lncRNAs and mRNAs

**Figure 4:**
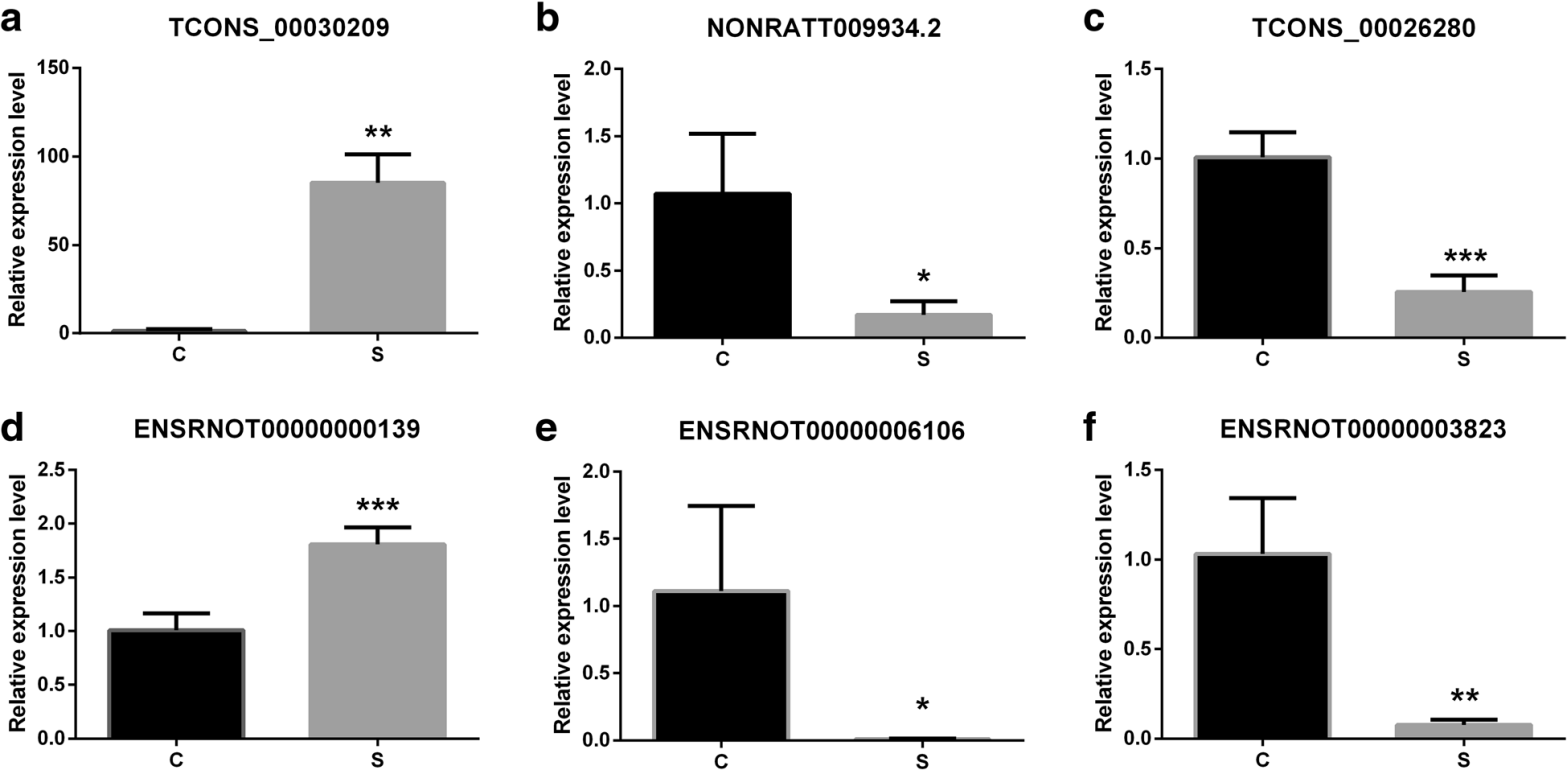
qRT-PCR validation of dysregulated DElncRNAs and mRNAs in urolithiasis rats compared with matched tissues of control rats. **a** expression level of lncRNA TCONS_00030209; **b** expression level of lncRNANONRATT009934.2; **c** expression level of lncRNATCONS_00026280; **d** expression level of mRNAENSRNOT00000000139; **e** expression level of mRNAENSRNOT00000006106; **f** expression level of mRNAENSRNOT00000003823. S represents urolithiasis rats, and **c** represents paired control rats. * represents *P* < 0.05; ** represents *P* < 0.01; and *** represents *P* < 0.001

Three lncRNAs and 3 mRNAs were chosen to verify the RNA-sequencing results in 4 pairs of samples by quantitative real-time PCR. The results showed that expression of lncRNA TCONS_00030209 was up-regulated, whereas NONRATT009934.2 and TCONS_00026280 were down-regulated. Meanwhile, of the 3 target mRNAs, ENSRNOT00000000139 was up-regulated, and ENSRNOT00000006106 and ENSRNOT00000003823 were down-regulated in kidney stone tissues compared with controls (Fig. 4). This result was consistent with the RNA sequencing. Hence, the finding provides valid evidence that these lncRNAs and mRNAs could be implicated in the pathogenesis of kidney stones.

### Delineation of GO and KEGG pathway analysis

**Figure 5:**
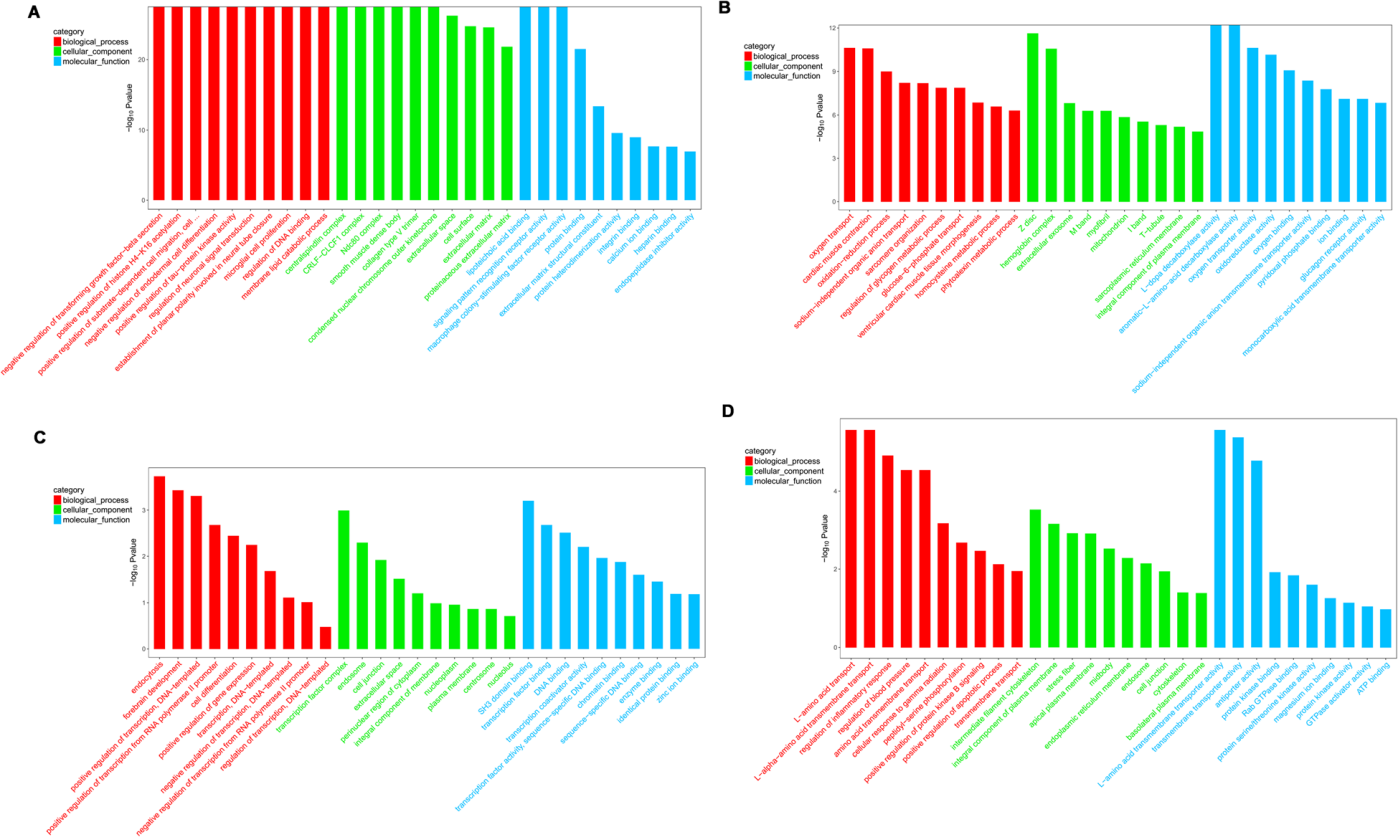
GO enrichment analysis for the mRNAs and circRNAs with the ten highest enrichment scores. **a** GO enrichment analysis for up-regulated mRNAs; **b** GO enrichment analysis for down-regulated mRNAs; **c** GO enrichment analysis for up-regulated circRNAs; **d** GO enrichment analysis for down-regulated circRNAs. Red bars are biological processes, green bars are cellular components, and blue bars are molecular functions. The ordinate is the -Log_10_ *P*-value (-LgP). Larger -Lg*P* values correlate with smaller *P*-values, indicating that the enrichment of differentially expressed genes/circRNAs in a given pathway is significant

**Figure 6:**
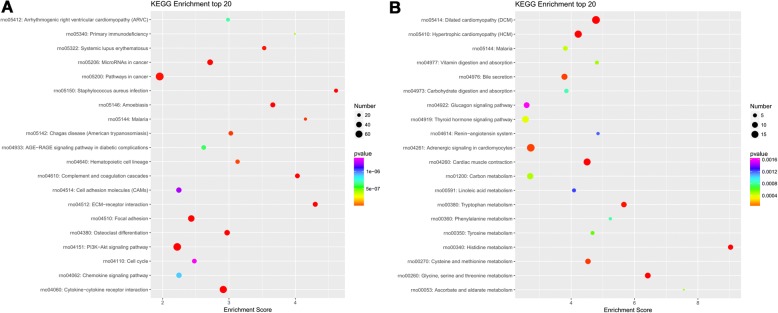
KEGG pathway enrichment analysis of the differentially expressed mRNAs with the twenty highest enrichment scores. **a** KEGG pathway enrichment analysis for up-regulated mRNAs; **b** KEGG pathway enrichment analysis for down-regulated mRNAs. The abscissa is the enrichment score. Size represents the number of enriched genes, and color indicates the degree of enrichment. Higher enrichment scores correlate with lower P-values, indicating that the enrichment of differentially expressed genes in a given pathway is significant

GO enrichment analysis of significant DEmRNAs can reveal the role of remarkably differentially regulated lncRNAs. In this study, we found that 2449 mRNAs were differentially expressed. GO enrichment analysis on the DEmRNAs showed that they were mainly related to biological processes and cellular components; only a few differentially expressed genes were associated with molecular function. Furthermore, those up-regulated mRNAs were obviously enriched in negative regulation of transforming growth factor-beta secretion, positive regulation of histone H4-K16 acetylation, positive regulation of substrate-dependent cell migration, cell attachment to substrate, centralspindlin complex, CRLF-CLCF1 complex, Ndc80 complex, lipoteichoic acid binding, signaling pattern recognition receptor activity, and macrophage colony-stimulating factor receptor activity of GO BP, CC and MF. The down-regulated mRNAs were enriched in oxygen transport, cardiac muscle contraction, the oxidation-reduction process, Z disc, hemoglobin complex, extracellular exosome, L-dopa decarboxylase activity, aromatic-L-amino-acid decarboxylase activity, and oxidoreductase activity (Fig. 5a, b). KEGG pathway enrichment analysis is devised for genes related to pathways and molecular interactions. The up-regulated mRNAs were enriched in cytokine-cytokine receptor interaction (rno04060), ECM-receptor interaction (rno04512), and complement and coagulation cascades (rno04610). Meanwhile, the down-regulated mRNAs were enriched in dilated cardiomyopathy (DCM) (rno05414), cardiac muscle contraction (rno04260), and histidine metabolism (rno00340) (Fig. 6).

GO analysis of differentially expressed circRNAs was performed to explore whether circRNAs regulate parental gene transcription. GO enrichment analysis of the circRNAs showed that they were mainly related to biological processes, and only a few differentially expressed genes were associated with cellular components and molecular function. In the up-regulated circRNAs, the GO terms for BP, CC, and MF were correlated with endocytosis, transcription factor complex, and SH3 domain binding. In the down-regulated circRNAs, the GO terms for BP, CC, and MF were correlated with L-amino acid transport, intermediate filament cytoskeleton, and L-amino acid transmembrane transporter activity (Fig. 5c, d).

### Co-expression of lncRNAs/mRNAs and function prediction

**Figure 7:**
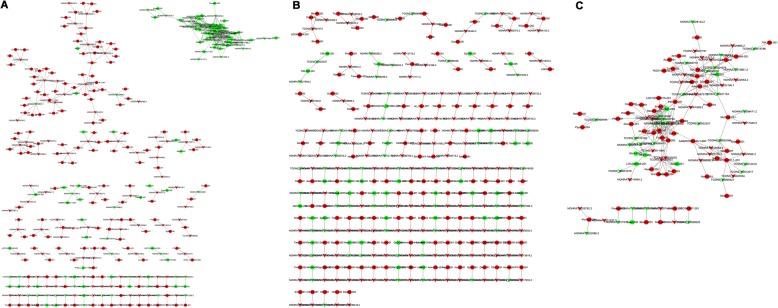
Construction of the lncRNA-mRNA co-expression network. **a** co-expression network of 129 DEmRNAs and 223 DElncRNAs; **b** LncRNAs and their potential cis-regulated nearby genes; **c** LncRNAs and their potential trans-regulated genes. Arrows represent DElncRNAs, and circular nodes represent DEmRNAs. Red indicates up-regulation, and green indicates down-regulation

To explore the molecular mechanisms of the pathogenesis of kidney stones, a co-expression network was built based on the expression levels of DElncRNAs and DEmRNAs. A total of 129 DEmRNAs and 223 DElncRNAs were involved in the network, and it consisted of 352 nodes and 500 edges (Fig. 7a). The top 5 up-regulated expressed DEmRNAs are Ephb6-201, Adprhl1-201, Arl5b-201, Ifitm6-201, and Svs1-201, and these mRNAs are associated with ephrin receptor activity, retrograde transport, ATP binding, GTP binding, ion binding, Rab GTPase binding, and ADP-ribosylarginine hydrolase activity. The DEmRNAs with down-regulated expression are Nebl-203, Klk1c9-202, Ndufb10-201, Rpl26-ps2-201, and Ldb3-203. These genes are implicated in a number of functions, such as actin filament binding, cytoskeletal protein binding, serine-type endopeptidase activity, mitochondrial respiratory chain complex ATP binding, ephrin receptor activity, RNA binding, and protein kinase C binding. LncRNAs and their potential cis-regulated adjacent genes are shown in Fig. 7b. Each lncRNA has a different number of nearby coding genes. For example, NONRATT004142.2 and TCONS_00001273 had a maximum number of 4 adjacent coding genes, while TCONS_00003675 only had 1 coding gene. LncRNAs and their potential trans-regulated genes are shown in Fig. 7c. TCONS_00009978 had a maximum number of 10 coding genes, whereas TCONS_00006475 only had 1 coding gene.

### Co-expression of circRNAs/miRNAs and function prediction

**Figure 8:**
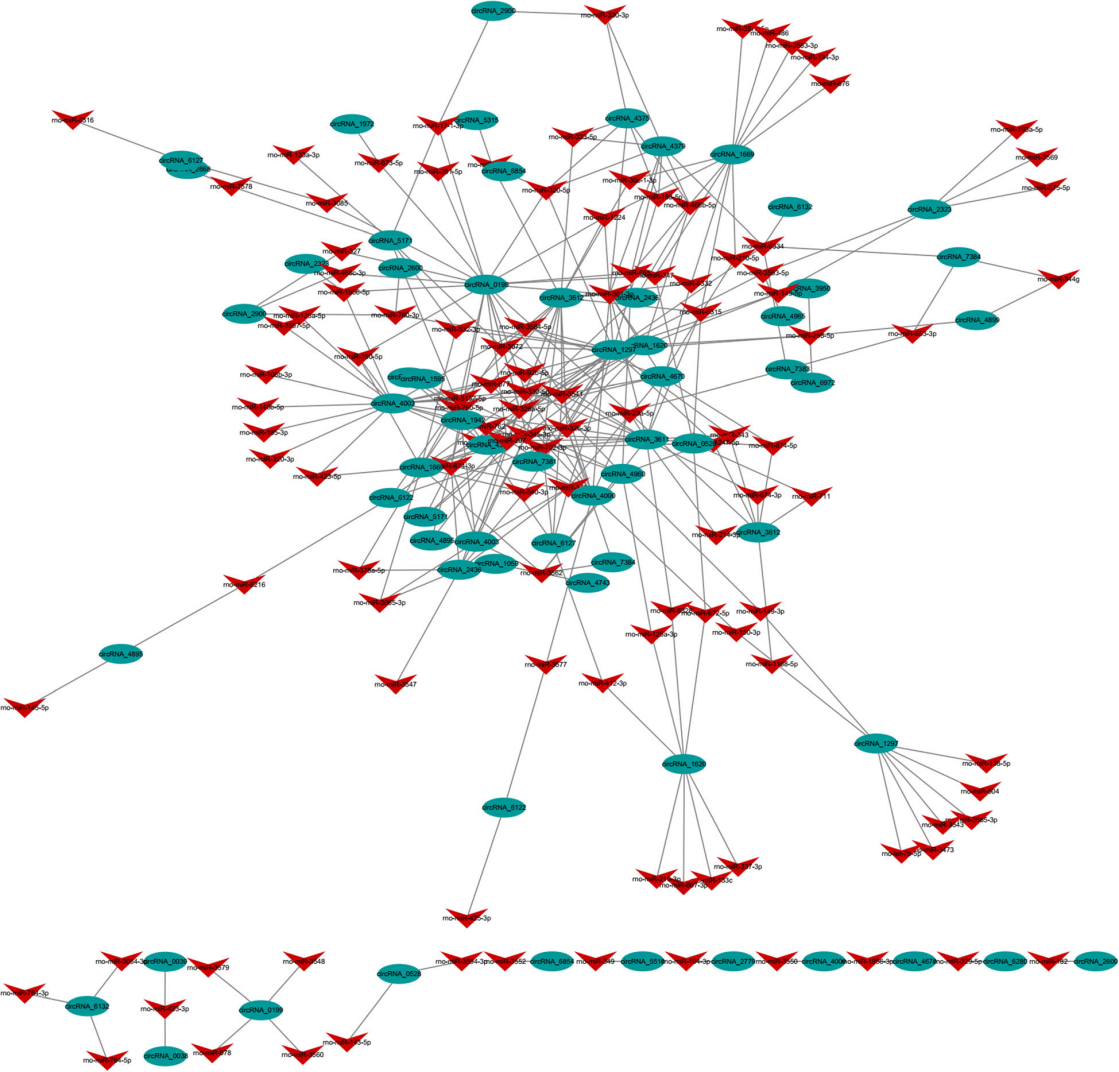
Construction of the circRNA-miRNA co-expression network. Oval nodes represent circRNAs, and arrow nodes represent miRNAs

All DEcircRNAs were predicted according to the complementary miRNA matching sequence. The network map was constructed with 64 circRNAs, 115 miRNAs and 300 relationships (Fig. 8). During the pathogenesis of kidney stones, there is a core circRNA-miRNA regulation network. The co-expression network revealed that different circRNAs have different numbers of predicted miRNAs. CircRNA_1297 established interactions with 23 miRNAs, while circRNA_1595 only established interactions with 1 miRNA.

## Discussion

Oral administration of ethylene glycol to rats for 4 weeks was reported to promote the deposition of crystals in the kidneys [12]. In our study, according to the results of urine parameters, serum parameters and histology, we conclude that 1% EG with drinking water successfully led to CaOx kidney stones and renal hypofunction. Conventional studies of gene regulation have focused on the protein-coding gene before the discovery of non-coding RNAs. Accumulating evidence showed that non-coding RNAs play critical roles in cellular functions [13]. However, comprehensive analyses of the profiles of differentially expressed lncRNAs and circRNAs in kidney stones have not yet been studied. To explore the potential function of lncRNAs and circRNAs in kidney stones, we performed expression profiles genome-wide for kidney stone and matched control tissues using RNA sequencing and bioinformatics analysis.

In our study, a total of 711 and 58 up-regulated, and 717 and 87 down-regulated lncRNAs and circRNAs, respectively, were identified to reveal the significant differential expression in kidney stones. Accordingly, 1732 up-regulated and 723 down-regulated mRNAs were identified in kidney stone tissues compared with controls. Among them, Ephb6 which located in the tubules of the outer medulla and cortex regulated cytoarchitecture of medullary tubule cells may affect the reabsorption ability of the kidney [14].

Arl5b is a trans-Golgi network-localized small G protein that plays a key role in regulating transport [15]. The podocyte cytoskeletal protein Nebl is associated with the dynamic changes of podocyte foot processes in podocyte injury [16]. DElncRNAs and mRNAs are distributed on all chromosomes. The expression levels of lncRNA (TCONS_00030209, NONRATT009934.2 and TCONS_00026280) and mRNA (ENSRNOT00000000139, ENSRNOT00000006106 and ENSRNOT00000003823) were verified by qRT-PCR, the results of which were consistent with high throughput sequencing. Co-expression networks of lncRNAs/mRNAs and circRNAs/miRNAs were constructed to predict the function of lncRNAs and circRNAs in kidney stone rats. Thus, our study provided a comprehensive understanding of the functions of lncRNAs and circRNAs in ethylene glycol-induced kidney stone rats; our findings could help determine the effects of lncRNAs and circRNAs on kidney stone pathology.

We performed GO enrichment analysis to describe gene attributes in biological processes, cellular components, and molecular functions related to dysregulated mRNAs. KEGG pathway analysis showed a significant change in cytokine-cytokine receptor interactions, ECM-receptor interactions, complement and coagulation cascades, dilated cardiomyopathy, cardiac muscle contraction, and histidine metabolism. It is well-known that the ECM-receptor interaction pathway was associated with tissue fibrosis and the androgen receptor mechanisms in nephrolithiasis [17], and renal ischemia/reperfusion injury [18].

In the present study, we found dysregulated lncRNAs in the kidney tissues of CaOx rats and predicted their corresponding mRNAs through cis- and trans-targeting. LncRNA NONRATT008306.2 and TCONS_00008586 were predicted to act on Rnf2 through cis-targeting. Rnf2 mediated transcriptional regulation by repressing the genes involved in development, differentiation, malignant transformation and cell cycle in human kidney cells [19]. Rnf2 was increased in the diabetic rat kidney and may play roles in the development of type 1 diabetes-induced renal fibrosis [20]. The function of Rnf2 in regulating basal and aldosterone-stimulated transcription of the α-ENaC gene (which was related to salt balance) was found in the duct cell line [21]. The lncRNA NONRATT006517.2, TCONS_00022796, NONRATT020511.2 and TCONS_00006475 were predicted to act on Xirp1 in a trans fashion. Xirp1 is an oxidative stress and antioxidant defense gene, and its expression increased in renal tissue under ischemia and reperfusion injury [22]. Xirp1 is a newly identified vitamin D receptor interacting protein and has an influence on VDR activity in the heart [23]. Xirp1 might play an important role in the pathology of kidney stones. However, few studies focus on the role of lncRNAs in kidney stones. In our study, most DElncRNAs in the co-expression network have not yet been annotated. It is worthwhile to perform further studies to reveal the underlying link between these lncRNAs and the pathomechanism of kidney disease.

GO enrichment analysis on the circRNAs showed that in the development of kidney stones, circRNAs were mainly related to biological processes. The function of DEcircRNAs was predicted according to the complementary miRNA matching sequence. A core circRNA-miRNA regulation network was present during the pathogenesis of kidney stones. Some of the predicted co-expressed miRNAs have been proven to be functional in hypercalciuria urolithiasis, such as rno-miR-138-5p co-expressed with circRNA_1297 and rno-miR-672-5p co-expressed with circRNA_0528 and circRNA_1620 [24].

The limitation of this study is that we just detected lncRNAs, circRNAs and miRNA changes but did notdid not validate at protein level. We did notdid not select areas of the rat kidney for RNA isolation, RT-PCR and sequencing analyses. We need to do further study to make more sense for mechanism of stone formation.

In conclusion, we found a profile of dysregulated lncRNAs, circRNAs and mRNAs that might be prospective clinical markers associated with the development of kidney stones. Our data laid a foundation for further potentially functional research into the lncRNAs and circRNAs involved in kidney stones. These results revealed that specific lncRNAs and circRNAs could be valuable for the diagnosis and therapy of kidney stones and may be of biological importance.

## Conclusion

The expression profile provided a systematic perspective on the potential functions of lncRNAs and circRNAs in the pathogenesis of nephrolithiasis and new targets for the prevention and treatment of kidney stones.

## Additional files

Additional file 1: **Table S1**. Statistical data of high-throughput sequencing for eight samples. Q30: the percentage of bases with quality value more than 30; 12,17,19,26: rats in CaOx group; 1,2,3,4: rats in control group. (DOCX 19 kb)

Additional file 2: **Table S2.** The mapped ratio of all samples. 12,17,19,26: rats in CaOx group; 1,2,3,4: rats in control group. (DOCX 18 kb)

## Abbreviations

DElncRNAs: Differentially expressed lncRNAs
DEmRNAs: Differentially expressed message RNAs
GO: Gene ontology
KEGG: Kyoto encyclopedia of genes and genomes database
lncRNAs: long noncoding RNAs
PCC: Pearson correlation coefficient
qRT-PCR: quantitative real-time polymerase chain reaction
