# Supplementary material for: Changing expression profiles of long non-coding RNAs, mRNAs and circular RNAs in ethylene glycol-induced kidney calculi rats
Source: BMC Genomics. 2018 Sep 10;19:660. doi: 10.1186/s12864-018-5052-8 (PMC6131827; doi:10.1186/s12864-018-5052-8)
Supplement: Supplementary file 1 — Table S1. Statistical data of high-throughput sequencing for eight samples. Q30: the percentage of bases with quality value more than 30; 12,17,19,26: rats in CaOx group; 1,2,3,4: rats in control group. (DOCX 19 kb) [file 12864_2018_5052_MOESM1_ESM.docx]

**Table S1 Statistical data of high-throughput sequencing for eight samples**

| Sample | Raw reads | Raw bases | Clean reads | Clean bases | Clean reads % | Clean bases % | Q30 |
| --- | --- | --- | --- | --- | --- | --- | --- |
| 12 | 98652338 | 14797850700 | 89912244 | 13440834690 | 91.14 | 90.83 | 97.68% |
| 17 | 98607590 | 14791138500 | 87704952 | 13106604080 | 88.94 | 88.61 | 97.82% |
| 19 | 98446250 | 14766937500 | 89167124 | 13326750617 | 90.57 | 90.25 | 97.81% |
| 26 | 98869984 | 14830497600 | 90045798 | 13457211897 | 91.07 | 90.74 | 97.80% |
| 1 | 98432650 | 14764897500 | 91813376 | 13724593446 | 93.28 | 92.95 | 97.59% |
| 2 | 98135566 | 14720334900 | 90735180 | 13554997953 | 92.46 | 92.08 | 97.43% |
| 3 | 98522728 | 14778409200 | 91464760 | 13669502767 | 92.84 | 92.50 | 97.43% |
| 4 | 98876246 | 14831436900 | 90718442 | 13558347336 | 91.75 | 91.42 | 97.56% |

Q30: percentage of bases with quality value above 30

12,17,19,26: rats in CaOx group

1,2,3,4: rats in control group
