# Supplementary material for: Changing expression profiles of long non-coding RNAs, mRNAs and circular RNAs in ethylene glycol-induced kidney calculi rats
Source: BMC Genomics. 2018 Sep 10;19:660. doi: 10.1186/s12864-018-5052-8 (PMC6131827; doi:10.1186/s12864-018-5052-8)
Supplement: Supplementary file 2 — Table S2. The mapped ratio of all samples. 12,17,19,26: rats in CaOx group; 1,2,3,4: rats in control group. (DOCX 18 kb) [file 12864_2018_5052_MOESM2_ESM.docx]

**Table S2: The mapped ratios of all samples**

| Sample | Total reads | Total mapped reads | Multiple mapped | Multiple mapped ratio (%) | Uniquely mapped | Uniquely mapped ratio (%) |
| --- | --- | --- | --- | --- | --- | --- |
| 12 | 89912244 | 87701276 | 10995824 | 12.23% | 76705452 | 85.31% |
| 17 | 87704952 | 85813053 | 11691329 | 13.33% | 74121724 | 84.51% |
| 19 | 89167124 | 87197085 | 11551679 | 12.96% | 75645406 | 84.84% |
| 26 | 90045798 | 87822345 | 11069535 | 12.29% | 76752810 | 85.24% |
| 1 | 91813376 | 89779085 | 12750445 | 13.89% | 77028640 | 83.90% |
| 2 | 90735180 | 88463804 | 10336830 | 11.39% | 78126974 | 86.10% |
| 3 | 91464760 | 88850557 | 15847445 | 17.33% | 73003112 | 79.82% |
| 4 | 90718442 | 88422086 | 12500315 | 13.78% | 75921771 | 83.69% |

12,17,19,26: rats in CaOx group

1,2,3,4: rats in control group
